# An association between multi-morbidity and depressive symptoms among Indian adults based on propensity score matching

**DOI:** 10.1038/s41598-022-18525-w

**Published:** 2022-09-15

**Authors:** Saurabh Singh, Neha Shri, Laxmi Kant Dwivedi

**Affiliations:** Department of Survey Research and Data Analytics, International Institute for Population Sciences, Mumbai, Maharashtra 400088 India

**Keywords:** Depression, Epidemiology

## Abstract

Keeping in view the cascade of disturbances caused by the co-existence of multi-morbidity and depression among aged population, this study aims to ascertain the independent impact of multi-morbidity as a risk factor for the development of depressive symptoms among adults living in India. The present study utilizes data from the nationally representative survey “Longitudinal Ageing Study in India” (LASI, Wave-1, 2017–2018). The eligible sample size was 62,244 adults aged 45 years and above. Descriptive statistics along with bivariate analysis was used to understand the prevalence of depressive symptoms. Further, binary logistic regression and Propensity Score Matching (PSM) methods were applied to examine the independent effect of multi-morbidity on depressive symptoms while controlling the selected background characteristics. Overall, around one-third respondents had at least one chronic disease and one-fifth had multi-morbidity. The most prevalent chronic disease reported in the sampled population was hypertension followed by diabetes and joint disease. It is observed that older adults with multiple chronic diseases had 77% higher odds of having depressive symptoms as compared to those without a history of chronic disease in the multivariable logistic regression model. Results obtained from PSM indicate that the risk of having depressive symptoms was 3.7% higher for adults with multi- morbidity. Depressive symptom was identified to be associated with a wider range of multiple physical health problems and people with multi-morbidity are at a higher risk of having depressive symptoms. It is imperative that multi-morbidity can be used as a screener for identifying people with depressive symptoms.

## Introduction

Multi-morbidity and depression pose serious challenges to the health systems particularly in low- and middle-income countries^1^. The presence of two or more chronic diseases at the same time in an individual termed as multi-morbidity^2^ which affects the majority of older individuals. On the other hand, depression being one of the leading causes of disability worldwide contributes majorly to the global disease burden^3^. According to the latest estimates published by National Statistical Office (NSO), India’s elderly population (aged 60 years and above) is projected to witness an increase of 41% over a decade in the period 2021–2031^4^. Furthermore, the growing epidemic of multi-morbidity in the global ageing population can not be ignored^5^.

Ageing is accompanied by the development of multiple chronic diseases and sometimes it affects the organs and tissues in a very different way. This results in failure of working of several organ systems resulting in the appearance of morbid conditions among older adults^6^. Under-recognized and untreated depression^7^, combined with other chronic diseases have a negative effect on overall individual health^8^. Epidemiological researches have highlighted the cascade of disturbances caused by the co-existence of multi-morbidity and depression in terms of disability and mortality^9,10^.

Although the mechanisms by which multi-morbidity affects depression are unclear, insights from the literature indicate a bidirectional relationship between depression and physical health disorders. People with multi-morbidity experience higher levels of disability, pain and cognitive impairment^11,12^ and tend to have a lower quality of life and higher rates of health care utilization^12^ contributing to the development of depression. Moreover, depressive symptoms induce negative health behaviour which in turn increases the risk of multi-morbidity^12^.

Studies have documented the relationship between depression and chronic disease such as diabetes, hypertension, coronary heart and lung diseases. Moreover, lower socio-economic status and less financially stable conditions push the aged person to be more vulnerable to mental health problems^13^. A follow-up study conducted in Hong Kong found that prevalent multi-morbidity is a significant predictor of depressive symptoms in adults^14^. The pathways through which the association between multi-morbidity and depression exist are mainly disability, pain, symptoms burden and loss of control over important aspects of life^15^.

In the recent years, low and middle-income countries with the least access to health care services, are expected to experience an increase in the burden of chronic diseases. Furthermore, India is experiencing an increasing burden of chronic non-communicable disease because of epidemiological transition^16^. Moreover, being a country with an increasing ageing population, the country will face an increased burden of aged population with morbidity. The main objective of this study is to ascertain the independent impact of multi-morbidity as a risk factor for the development of depressive symptoms among adults living in India. Further, this study also aims to understand the patterns of depressive symptoms in relation to the selected demographic and socio-economic conditions.

## Methods

### Data

The present study utilizes data from first wave of the nationally representative survey “Longitudinal Ageing Study in India” (LASI, WAVE-I, 2017–2018) conducted under the stewardship of the Ministry of Health and Family Welfare, Government of India, coordinated by the International Institute for Population Sciences (IIPS), Mumbai.

This survey adopts a multistage stratified area probability cluster sampling design and a three-stage and four-stage sampling design used in rural and urban areas respectively. The first stage was selection of sub-districts (Tehsils/Talukas), and then selection of villages in rural areas and wards in urban areas in the selected sub-districts. In third stage, fixed number of households (i.e. 32) were selected from each selected villages in rural area while Census Enumeration Block (CEB) was randomly selected in each urban ward then fixed number of households (i.e. 35) were selected from each CEB in Urban areas.

LASI provides information for Indian states and union territories on demographics, household economic status, chronic health conditions, symptoms-based health conditions, functional health, mental health (cognition and depression) and other components of the older adults in India. In our present study, Individual file was used for this study. As part of the ethics protocols, individual and household informed consent forms were used in the survey. Consent for blood samples collection and storage and proxy consent were also taken and participants were provided referral letters and biomarker report cards if their health measurements were outside the normal range. Based on the objectives, respondents aged 45 years and older were considered eligible for the study. Individuals who reported having a neurological/psychiatric problem were excluded from the study. The eligible sample size for the study was 62,244. The estimates were derived after assigning the sampling weights. Individuals aged less than 45 years and those with incomplete information were excluded from the analytical sample.

### Description of variables

#### Outcome variable

In the present study, the outcome variable is depressive symptoms. Depressive symptoms were assessed using a shortened set of ten symptomatic questions based on Centre for Epidemiologic Studies Depression Scale (CES-D) originally developed by Radloff^17^. The 10 items used for assessment of depression included seven negative symptoms (trouble concentrating, feeling depressed, low energy, fear of something, feeling alone, bothered by things, and everything is an effort), and three positive symptoms (feeling happy, hopeful, and satisfied)^18^. Response options included rarely or never (< 1 day), sometimes (1 or 2 days), often (3 or 4 days), and most or all of the time (5–7 days) in a week prior to the interview. For negative symptoms, rarely or never (< 1 day), and sometimes (1 or 2 days) were scored zero, and often (3 or 4 days) and most or all of the time (5–7 days) categories were scored one. Scoring was reversed for positive symptoms. The overall score ranges from zero to ten and scores of four or more were used to calculate the prevalence of depressive symptoms. It was further recoded into dichotomous variable using the CES-D scale as 0 “not having depressive symptoms” and 1 “have depressive symptoms”^18^.

### Predictor variables

Multi-morbidity was the main predictor variable as per the objectives of this study. To assess the existence of a morbid condition, information was collected on nine self-reported diagnosed chronic health conditions. The prevalence of chronic health conditions/diseases was assessed based on ever diagnosed by health professionals such as MBBS, MD, BDS, and AYUSH only. Participants were asked if they have ever been diagnosed/told to be having a particular disease by any health professional. Morbid condition was calculated using nine chronic diseases. These diseases were hypertension, diabetes, cancer, chronic lung disease, chronic heart disease, stroke, chronic bone/joint disease and high cholesterol. Respondents were classified to be multi-morbid depending on the presence of multiple chronic diseases at the time of survey. The response were recoded as 0 “no morbidity”, 1 “single morbid condition”, 2 “two morbid condition” and 3 “three or more morbid condition”. It was further classified into dichotomous variable by combining 0 and 1 as 0 “not multi-morbid” and combining 2 and 3 coded as 1 “multi-morbid”. In propensity score matching analysis, this variable acts as a treatment variable.

#### Control variables

Respondents were categorized as men and women on the basis of sex. The age was categorized as 45–59 years, 60–69 years, 70–79 years and 80 years and above. Based on the information of current marital status, respondents were categorized as currently married and second category include widowed/divorced/separated/single. The participants were asked if they ever consumed tobacco and alcohol and their responses were categorized as 0 “never” and 1 “ever”. Years of schooling was recoded as 0 “no formal education”, 1 “1–5 years of schooling”, 2 “6–9 years of schooling” and 3 “10 or more years of schooling”. The wealth status of the respondents was assessed using household consumption data on expenditures on food and non-food items which were further divided into five quintiles. The wealth quintiles were recoded as “poor” for individuals in ‘poorest & poorer’ wealth quintile, “middle” and “rich” for individuals in ‘richer and richest’ wealth quintile. The general health status was measured using self-rated health (SRH) question. The respondents were asked how they would say their health status is in general. The possible responses were categorised into three groups as follows: good (‘very good’ and ‘good’), fair (moderate), and poor (‘poor’ and ‘very poor’). The place of residence was recoded as rural and urban. The region where respondents currently live was coded as North, Central, East, Northeast, West, and South. Religion was coded as Hindu, Muslim, Christian, and Others. Caste was recoded as Scheduled Tribe (ST), Scheduled Caste (SC), Other Backward Class (OBC), and others. The living arrangement of the respondents were categorized as “living alone”, “living with spouse and with children” and ‘others” which includes other family members/relatives.

### Statistical analysis

Descriptive statistics along with bivariate analysis was used to understand the distribution of sample characteristics and prevalence of depressive symptoms among study population. Appropriate sampling weight was applied while carrying out univariate and bivariate analysis to compensate for unequal selection probabilities at various levels of selection and to compensate for non-response. Further, binary logistic regression analysis has been employed to fulfil the objective of the study. The results were presented in the form of adjusted odds ratio (OR) and unadjusted odds ratio (OR) with a 95% confidence interval (CI).

The study uses propensity score matching to assess the impact of multi-morbidity on those who have depressive symptoms. In our study, we select the multi-morbidity as the treatment status and the confounding factors as the observed baseline characteristics. Using the propensity scores, cases were individually matched to controls using the nearest neighborhood matching approach. This is the counterfactual model to compute the effect of multi-morbidity on depressive symptoms while controlling the selected background characteristics and biases arise due to non-random assignment of subjects in the treatment and control group^19^. All the statistical analysis were performed using STATA 14.0 software. All p values were two-tailed and difference was defined to be significant when *p* < 0.05.

### Declarations

This study uses a secondary data set and humans were involved in this study. All the methods were carried out in accordance with relevant guidelines and regulations and ethical approval was taken from Indian Council for Medical research (ICMR) for conducting the survey. Informed consent was obtained from all the participants prior to the interview.

## Results

The unweighted frequency and weighted percentage distribution of the study sample (older adults age 45 years and above) by various socio-economic status and chronic health-related problems are shown in Table 1. The sampled consisted of 45.6% male and 54.4% female. Approximately half of the eligible respondents(50.4%) were aged 45–59 years, 30% were aged 60–69 years, 20% were aged 70 years and above. A majority of the respondents were currently married (74.1%), and a quarter were widowed/divorced/separated or never married. A major proportion of the respondents never smoked tobacco and consumed alcohol (62.9% and 84.9% respectively). One-half of the sample did not receive any formal education (50.7%), while 17% had 1 to 5 years of education, 14% had 6–9 years of education, and a little less than one-fifth 17.9% had 10 or more years of education. A little less than two-fifth (38.2%) of the respondents reported their health as “good” while almost 44% reported their health to be “fair” and 18% reported their health being “poor”. Sixty nine percent of the respondents resided in rural area and a majority of the eligible population followed “Hinduism”. Majority of the respondents were from “OBC” category (45.4%), one-fifth were from SCs (19.3%) and around nine percent belonged to STs. Of the total study population, 3.7% of older adults aged 45 and above were living alone, 72.6% of the study population lived with their spouse, and 24% of the study population lived with people other than spouse. Hypertension was the most prevalent chronic disease (27%) in the study population, followed by joint disease and diabetes (16% and 12% respectively). Further, around 6% reported having chronic lung disease and 3.70% suffered from chronic heart disease. Moreover, 0.62% had cancer and 1.19% had high cholesterol. About 27.2% of adults aged 45 and above suffered from at least one chronic disease, 12.6% had two diseases simultaneously while 5% of the study population had three or more disease at the same time.

**Table 1:** Background characteristics of study population, LASI Wave 1 (2017–2018).

Variable	Labels	Frequency	Percent	Depressive symptoms
Sex	Male	28,837	45.57	25.16
	Female	33,407	54.43	29.96
Age at last birthday	45–59	32,707	50.35	25.94
	60–69	18,062	29.62	28.51
	70–79	8504	14.78	30.60
	80+	2971	5.25	33.27
Marital status	Currently married	46,977	74.14	25.29
	Widowed/divorced/separated	15,267	25.86	34.88
Tobacco smoke	Never smoked	39,449	62.93	27.77
	Ever smoked	22,795	37.07	27.78
Consume alcohol	Never	51,014	84.86	28.16
	Ever	11,230	15.14	25.60
Years of schooling	No formal education	29,188	50.69	31.61
	1–5 years of education	11,386	17.37	26.92
	6–9 years of education	9859	14.08	23.76
	10 and above years of education	11,811	17.87	20.89
Wealth quintile	Poor	24,860	42.40	28.45
	Middle	12,519	20.46	28.95
	Rich	24,865	37.14	26.35
Self-rated health	Good	26,001	38.15	20.39
	Fair	25,983	44.10	28.04
	Poor	10,260	17.75	42.96
Place of residence	Rural	40,425	69.05	28.54
	Urban	21,819	30.95	26.05
Region	North	11,437	12.52	25.64
	Central	8447	18.14	28.39
	East	10,463	21.39	28.24
	North–East	6260	1.18	15.14
	West	8244	19.74	33.55
	South	17,393	27.03	24.31
Religion	Hindu	45,681	82.20	28.02
	Muslim	7358	11.26	29.57
	Christian	6257	3.02	23.41
	Others	2948	3.52	19.92
Caste	SC	10,422	19.28	31.37
	ST	10,915	8.68	25.61
	OBC	23,376	45.39	28.11
	Others	17,531	26.65	25.29
Living arrangement	Living alone	2169	3.66	44.02
	Living with spouse	45,765	72.64	25.30
	With other than spouse	14,310	23.71	32.83
Chronic conditions	Hypertension	17,681	27.19	30.34
	Diabetes	7916	12.25	27.11
	Cancer	407	0.62	32.62
	Chronic lung disease	3425	6.24	35.13
	Chronic heart disease	2188	3.70	28.51
	Stroke	984	1.71	41.80
	Joint disease	8824	15.45	33.61
	High cholesterol	2144	1.19	23.58
Number of chronic morbidity	No morbidity	34,042	55.10	25.69
	Only one morbidity	17,067	27.21	28.91
	Only two morbidity	7888	12.56	32.77
	3 or more morbidity	3247	5.12	31.94
Total		62,244		27.77

Total, N = 62,244, percentage distribution are weighted.

The prevalence of depressive symptoms was higher among women than men (30% among women vs 25% among men). The proportion of respondents having depressive symptoms increased with higher ages. For instance, around 25.9% of respondents aged 45–59 years were depressed, which rose to 30.6% and 33.3% among those aged 70–79 years and 80 years and above respectively. Prevalence of depressive symptom was higher among those widowed/divorced/separated/never married. Further, the prevalence of depressive symptoms was approximately same i.e., 28% among tobacco users and non-users. The prevalence of depressive symptoms decreased with increase in years of schooling. For instance, one-third (31.6%) of those without any formal education were depressed while 21% of respondents with 10 or more years of schooling had depressive symptoms. The prevalence of depressive symptoms did not vary much by wealth quintile. Moreover, the prevalence of depressive symptoms was highest among those who reported their health to be “poor” (43%). The prevalence of depressive symptoms was 26% and 29% respectively among those residing in urban and rural area. The highest prevalence of depressive symptoms was observed in western region (34%) while the lowest was observed in North-Eastern region (15%). The highest prevalence of depressive symptoms was observed among those living alone (44%). The prevalence of depressive symptoms was highest among those suffering from stroke (41.8%) and chronic lung disease (35.1%), followed by joint disease (33.61%) and cancer (32.62%). Surprisingly, around one fourth (26%) of the respondents without any morbid condition had depressive symptoms. Moreover, the proportion of respondents with depressive symptoms increased with an increase in the number of morbid conditions.

Tables 2 and 3 presents the odds ratios obtained from logistic regression analysis to determine the effect of chronic health conditions on depressive symptom among adults in India. Model 1 presents the association between chronic health conditions and depressive symptoms whereas, the association persisted in Model 2 which is adjusted for socio-economic and demographic characteristics. In the first model, respondents with one morbid condition have 1.20 times higher odds of experiencing depressive symptoms in comparison to those without any morbid condition. Further, respondents with two morbid conditions and three or more co-morbid conditions were 1.35 times odds and 1.54 times odds more likely to have depressive symptoms in reference to those without any morbidity.

**Table 2:** Odds ratio and 95% CI for various factors associated with depressive symptoms among older adults in India (LASI wave 1, 2017–2018).

Variables	Label	Model 1			Model 2		
		Unadjusted odds ratio			Adjusted odds ratio		
		OR	CI		OR	CI	
Number of chronic morbidity	No morbidity						
	Only one morbidity	1.20***	1.16	1.26	1.09***	1.04	1.14
	Only two morbidity	1.35***	1.28	1.43	1.16***	1.09	1.23
	3 or more morbidity	1.54***	1.42	1.66	1.23***	1.13	1.34
Sex	Male^®^						
	Female				0.96*	0.91	1.00
Age	45–59^®^						
	60–69				0.97	0.93	1.01
	70–79				0.99	0.94	1.05
	80 +				1.04	0.95	1.14
Marital status	Currently married^®^						
	Widow/divorced/single				1.49***	1.29	1.72
Smoking	Never smoked^®^						
	Ever smoked				0.95**	0.91	1.00
Alcohol	Never^®^						
	Ever				0.93**	0.88	0.99
Years of schooling	No formal education^®^						
	1–5 years of education				0.83***	0.79	0.88
	6–9 years of education				0.80***	0.75	0.85
	10 and above years of education				0.69***	0.65	0.73
Wealth quintile	Poor^®^						
	Middle				0.91***	0.86	0.95
	Rich				0.89***	0.85	0.93
Self-rated health	Good^®^						
	Fair				1.41***	1.35	1.47
	Poor				2.44***	2.31	2.58
Residence	Rural^®^						
	Urban				0.92***	0.88	0.96
Region	North^®^						
	Central				0.98	0.91	1.05
	East				1.01	0.95	1.08
	North–East				0.57***	0.52	0.63
	West				1.36***	1.27	1.46
	South				0.89***	0.84	0.95
Religion	Hindu^®^						
	Muslim				0.97	0.91	1.03
	Christian				1.11**	1.02	1.20
	Others				0.63***	0.57	0.70
Caste	SC^®^						
	ST				0.79***	0.74	0.85
	OBC				0.91***	0.87	0.96
	Others				0.88***	0.83	0.94
Living arrangement	Living alone^®^						
	Living with spouse				0.74***	0.63	0.88
	With other that spouse				0.66***	0.6	0.73

Note: ***p<0.01, **p<0.05, *p<0.1

**Table 3:** Unadjusted Odds ratio and 95% CI for various chronic diseases associated with depressive symptoms among older adults in India (LASI Wave 1, 2017–2018).

Variables	Label	Unadjusted Odds ratio	95% CI	
Hypertension	No^®^			
	Yes	1.14***	1.09	1.19
Diabetes	No^®^			
	Yes	0.93***	0.88	0.99
Cancer	No^®^			
	Yes	1.49***	1.21	1.84
Chronic lung disease	No^®^			
	Yes	1.46***	1.36	1.57
Chronic heart disease	No^®^			
	Yes	1.18***	1.07	1.30
Stroke	No^®^			
	Yes	1.66***	1.45	1.89
Bone disease	No^®^			
	Yes	1.46***	1.39	1.53
High cholesterol	No^®^			
	Yes	0.76***	0.68	0.84

® represents reference category.

*Represents statistically significant values at 90% CI.

**Represents statistically significant values at 95% CI.

***Represents statistically significant values at 99% CI.

After adjusting with other variables, risk of having depressive symptoms increased with increase in number of diseases. For instance, having one and two chronic morbidity increased the risk of having depressive symptoms by 1.09 times odds and 1.16 times odds respectively in comparison to those without any chronic morbidity. Similarly, respondents with three or more comorbid condition were 1.23 times odds significantly more likely to have depressive symptoms than those without any disease. Interestingly, females were at less risk of having depressive symptoms than men. However, increasing age did not show statistically significant association with risk of developing depressive symptoms. Unsurprisingly, widowed/divorced/separated/never married were at 1.5 times odds higher risk of depressive symptoms than currently married. Population who ever smoked and ever consumed alcohol were less likely to have depressive symptoms. Increasing years of schooling and higher wealth quintiles were associated with lesser risk of having depressive symptoms. Having “poor” self-rated health increased the risk of depressive symptoms by 2.44 times odds in reference to those with “good” health. Urbanites are found to be eight percent less likely to have depressive symptoms than those residing in rural area. People residing in North-east region and south were 43 percent and 11 percent respectively less likely of having depressive symptoms in reference to residing in northern region. Living with spouse decreased the risk of developing depressive symptoms by 26 percent while leaving with other but not with spouse decreased the risk of having depressive symptoms by 34 percent than living alone. Results from Table 3 show that chronic condition such as hypertension, cancer, lung disease, heart disease, stroke and bone disease were statistically significantly associated with depressive symptoms and having any of these morbid conditions increased the risk of having depressive symptoms. Surprisingly, having diabetes and high cholesterol reduced the risk of having depressive symptoms and results of table 3 are obtained without adjusting the selected covariates.

Table 4 illustrates the results obtained from propensity score matching analysis. Most of the bias attributable to observable covariates can be eliminated by using Propensity Score Matching. The final number of blocks were 17. The unmatched sample estimate presents the raw estimates i.e. without matching result shows that those older adults who have multi-morbidity had 5.4% higher chance to have depressive symptoms than older adults who did not have multi-morbidity. ATT, ATU and ATE show the estimates after matching. Using the nearest neighbour matching with replacement method, the average treatment effect on the treated (ATT) values among treated and controls were 0.295 and 0.290 which means that if the multi-morbidity was not present among those adults who have multi-morbidity, the prevalence of depressive symptoms would have been less. ATU values in treated and control groups were 0.242 and 0.286 respectively, which means if older adults who do not have multi-morbidity currently develop multi-morbidity, their chance of having depressive symptoms will increase by 4.3%. Average treatment effect (ATE) shows the difference between the treated and the untreated which was 0.037, which means on an average, there is a 3.7% higher chance of having depressive symptoms for multi-morbid adults.

**Table 4:** The Effect of Multi-Morbidity and different chronic diseases on Depression, Analysis through Propensity Score Matching.

Variable	Sample	Treated	Controls	Difference	SE	T-stat
Depressive symptoms	Unmatched	0.295	0.242	0.054	0.005	11.850
	ATT	0.295	0.290	0.006	0.010	0.540
	ATU	0.242	0.286	0.043		
	ATE			0.037		

*ATT* average treatment effect on the treated, *ATU* average treatment effect on the untreated, *ATE* average treatment effect.

**Figure 1:**
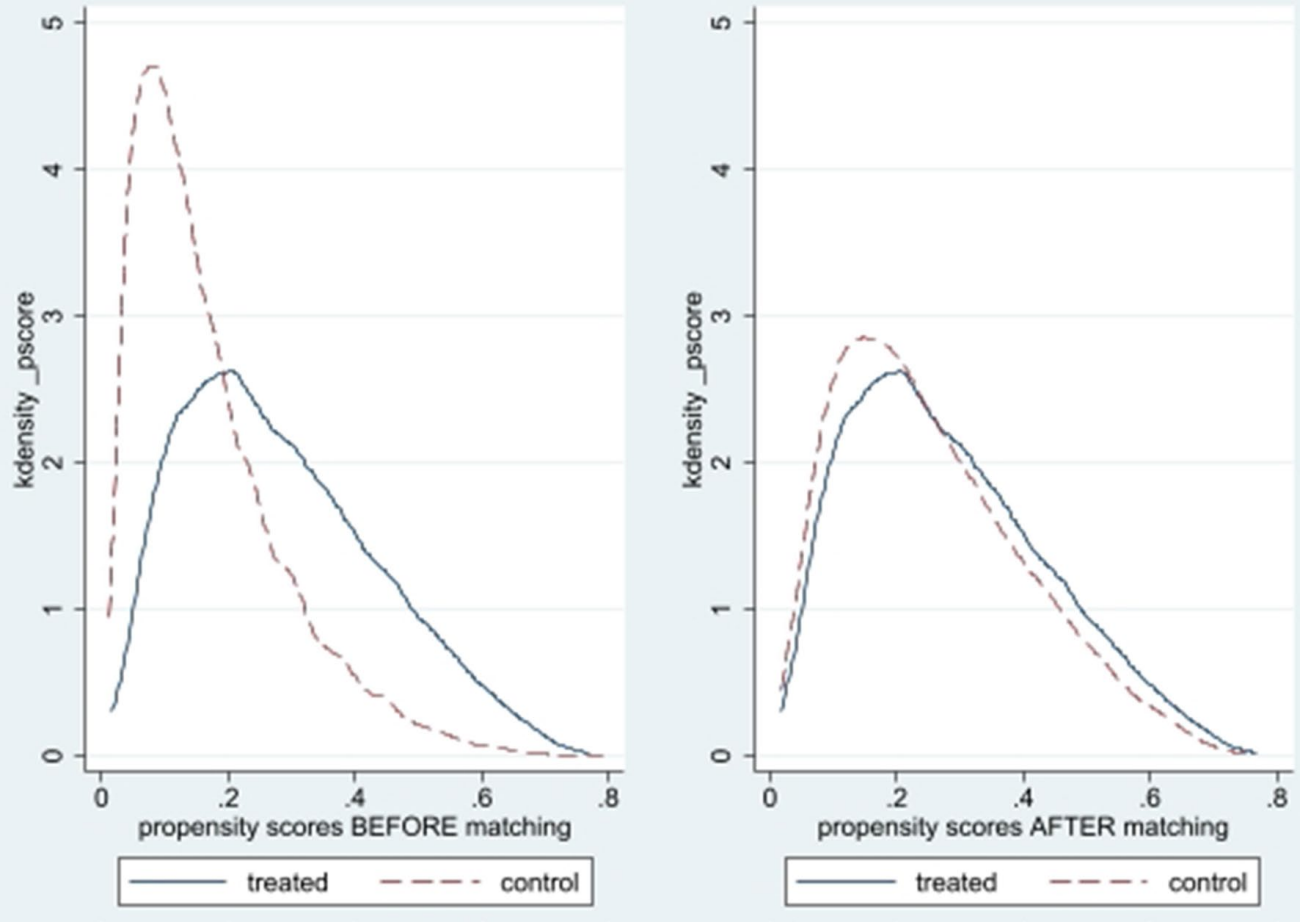
Balance plot.

**Figure 2:**
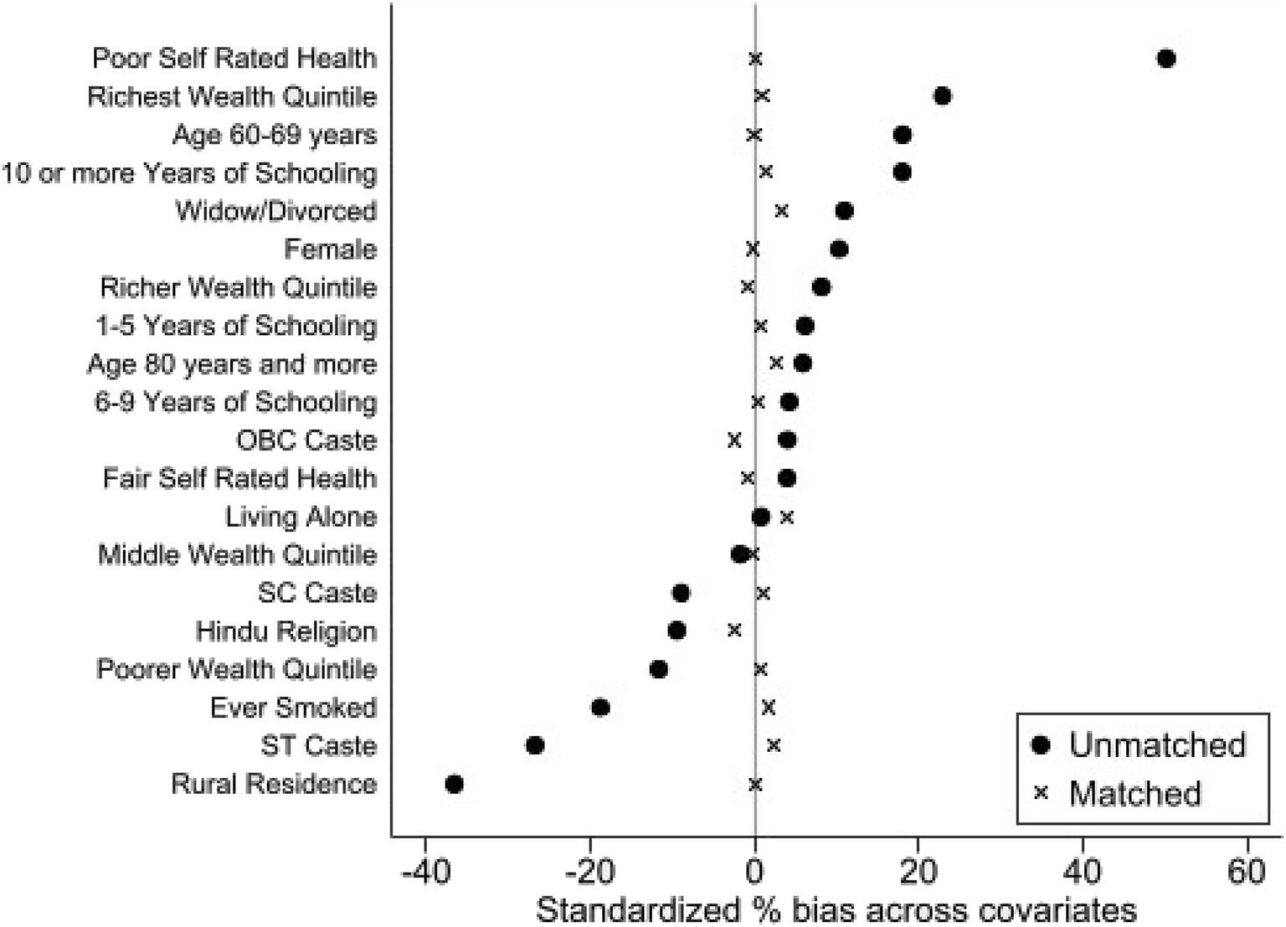
Love plot.

Common support improves the quality of matching by discarding individuals in which there is more availability of merged samples. Table 5 demonstrates that the number of dropped elders due to common support was minimal. This also reveals that while comparing individuals not having depressive symptoms with individuals having depressive symptoms, 355 samples were discarded (all from the untreated group) from the sample of 62,244 observations. The balance plot of the covariates of the treatment and control group before and after matching cases has been shown in Fig. 1. It indicates that both the control and treatment groups were balanced indicating the unbiasedness in the estimated treatment effects. Love plot indicates standardized % bias across all covariates in matched and unmatched sample (Fig. 2).

**Table 5:** Common support.

Treatment assignment	Common support		Total
	Off support	On support	
Untreated	355	50,754	51,109
Treated	0	11,135	11,135
Total	355	61,889	62,244

## Discussion

In this study, the prevalence and correlates of depressive symptoms with an emphasis on multi-morbidity among Indian adults was assessed. Overall, a little less than one-third of the sample respondents had at least one-chronic disease and almost one-fifth had multi-morbidity. A higher level of depressive symptoms was observed among those having chronic lung disease, followed by joint disease, cancer and hypertension. Overall, 33% of the respondents with two morbid condition and 32% with three or more co-morbidity had depressive symptoms.

Our results suggest that the risk of having depressive symptoms among adults is significantly related to multi-morbidity^12^. Older adults with three or more chronic disease had 54% higher chance of having depressive symptoms as compared to those without a history of chronic disease in the unadjusted model. Moreover, after adjusting the socio-economic and demographic characteristics, individuals with multiple chronic disease had 23% higher odds of having depressive symptoms as compared to those without a history of chronic disease. A number of previous studies have reported the relationship between multi-morbidity and depressive symptoms^20,21^. Results indicated that with the increase in the number of morbid condition, the risk of developing depressive symptoms increased significantly. Similar to our findings, You and colleagues have also reported that elderly with three or more chronic disease were more likely to have depressive symptoms^22^. A review published in 2019 demonstrate that there exists an interaction between various disease in an individual resulting in physical and cognitive decline^23^. Further, this physical and cognitive impairment increases the severity and burden of multi-morbidity constituting a vicious circle^23^. This study reports lower level of depressive symptom among females as compared to males which is augmented by the theory that male who survive are healthier than women^24^. However, Freidrich et al. have reported higher depression levels among females than males due to the change in hormonal levels as a result of menopause^25^.

Contextually, it is important to understand the impact of social determinants of health in studying morbidity and depressive symptoms. Those who reported their health to be poor were 2.44 times odds more likely to have depressive symptoms than those who reported their health to be good. Previous studies have shown that multimorbidity alters the physical status of an individual which impacts their overall health status. Further, people with depressive symptoms have a poorer quality of life than those with chronic health conditions but not depressive symptoms^26^. Further, the bi-directional relationship between multi-morbidity and poor health status further exacerbates its impact on depressive symptoms^11,12,26^. However, the interpretation of poor health status, multi-morbidity and symptoms of depression is cautious, as the cross-sectional study may have a cohort effect on it. We could infer from the findings that adults residing in rural area were more likely to have depressive symptoms which were consistent with study of You and colleagues^22^. Moreover, the cultural differences between different regions of the country also have a significant impact on depression. Results show that people from North-Eastern and Southern India, were significantly less likely to have depressive symptoms than people residing in North. Kulkarni and his colleagues also found similar evidences of lower depression levels in north–east and southern part of the country^27^. However, people residing in west were more likely to have depressive symptoms in reference to respondents from Northern India. Region-wise differences in the levels of depressive symptoms is attributed to unmeasurable contextual factors. Living with spouse or someone else decreased the risk of having depressive symptoms among Indian adults.

Chronic condition such as hypertension, cancer, lung disease, heart disease, stroke, bone disease and psychiatric problem were observed to be a significant predictor of depressive symptoms in our study. A number of studies have confirmed that multimorbidity increase the risk of mental health disorders^2,28,29^.

One of the plausible reason behind this could be that more disease requires more visit to health care systems and longer duration in hospitals which negatively affects the mental status of an individual. Disease such as diabetes, stroke, thyroid disorder causes pathophysiological changes in brain or immune functions which adversely affects depression^30^. On the other hand, studies have reported the protective role of Metformin used in treating diabetes which ameliorates depression^31,32^. Metformin helps in reducing depression by improving cognitive performance and glucose metabolism^33^. In line with the previously established notion of protective role of diabetes for depression, we found that those with diabetes had reduced risk of having depression. Results obtained from PSM indicate that the risk of having depressive symptoms is 3.7% higher for multi-morbid adults.

While interpreting our results, there are several limitations which should be noted. First, only association between multi-morbidity and depressive symptoms can be inferred from our results not the causality as the data used in this study is cross-sectional in nature (being first wave of the longitudinal survey). This means that data includes exposure and outcome concurrently. In PSM analysis, the probability of having the risk of depressive symptoms might increase if individuals who are having only one morbidity is excluded from the category of without morbidity. However, study highlights the association between multi-morbidity and depressive symptoms. The presence or absence of multi-morbidity is based on self-reported measures which might lead to confounding and may not capture the actual prevalence of disease due to low awareness of symptoms of many health conditions, inadequate diagnosis^34^. Although total scores do not correspond to clinical diagnosis of depression, but they indicate the level of high depressive symptoms which can be of clinical relevance^35^. Despite the limitations, since, the study utilizes the national representative data, the findings obtained can be generalized.

## Conclusion

To conclude, we have identified that depressive symptoms is associated with a wider range of multiple physical health problems and people with multi-morbidity are at the higher risk of having depressive symptoms. The association between multi-morbidity and depressive symptoms was consistent after adjusting for confounding factors. These findings have important implications for the management of depression and chronic morbidity in health care settings especially at a time when India is witnessing a rise in the ageing population. Moreover, multi-morbidity can be used as a screener for identifying people with depressive symptoms (Supplementary Information).

## Supplementary Information

Supplementary Information.

## Data Availability

This study uses secondary data which is publicly available on request to IIPS, Mumbai through https://www.iipsindia.ac.in/content/lasi-wave-i.
