# Supplementary material for: An association between multi-morbidity and depressive symptoms among Indian adults based on propensity score matching
Source: Sci Rep. 2022 Sep 15;12:15518. doi: 10.1038/s41598-022-18525-w (PMC9478135; doi:10.1038/s41598-022-18525-w)
Supplement: Supplementary file 1 — Supplementary Information. [file 41598_2022_18525_MOESM1_ESM.docx]

**An association between multi-morbidity and depressive symptoms among Indian adults based on propensity score matching**

*Saurabh Singh, Neha Shri*, Dr. Laxmi Kant Dwivedi*

*Corresponding author

**Appendix**

Table S1. Correlation matrix of covariates used in the logistic regression analysis

|  | Depressive  symptoms | chronic  disease | Sex | Age | Marital  Status | Smoking | Alcohol | Years of  Schooling | Wealth  quintile | SRH | Residence | Region | Religion | Caste | Living Arrange-  ment |
| --- | --- | --- | --- | --- | --- | --- | --- | --- | --- | --- | --- | --- | --- | --- | --- |
| Depressive  symptoms | 1.00 |  |  |  |  |  |  |  |  |  |  |  |  |  |  |
| Chronic  disease | 0.06 | 1.00 |  |  |  |  |  |  |  |  |  |  |  |  |  |
| Sex | 0.05 | 0.06 | 1.00 |  |  |  |  |  |  |  |  |  |  |  |  |
| Age | 0.06 | 0.15 | -0.03 | 1.00 |  |  |  |  |  |  |  |  |  |  |  |
| Marital Status | 0.09 | 0.06 | 0.28 | 0.31 | 1.00 |  |  |  |  |  |  |  |  |  |  |
| Smoking | -0.01 | -0.09 | -0.37 | 0.04 | -0.06 | 1.00 |  |  |  |  |  |  |  |  |  |
| Alcohol | -0.03 | -0.06 | -0.39 | -0.03 | -0.10 | 0.37 | 1.00 |  |  |  |  |  |  |  |  |
| Years of Schooling | -0.09 | 0.10 | -0.30 | -0.16 | -0.19 | -0.05 | 0.05 | 1.00 |  |  |  |  |  |  |  |
| Wealth quintile | -0.03 | 0.13 | -0.01 | -0.03 | -0.04 | -0.06 | -0.01 | 0.22 | 1.00 |  |  |  |  |  |  |
| SRH | 0.16 | 0.28 | 0.08 | 0.21 | 0.12 | 0.01 | -0.03 | -0.11 | 0.00 | 1.00 |  |  |  |  |  |
| Residence | -0.03 | 0.17 | 0.01 | -0.03 | 0.01 | -0.14 | -0.07 | 0.32 | 0.00 | -0.03 | 1.00 |  |  |  |  |
| Region | 0.00 | 0.09 | 0.01 | 0.00 | 0.03 | -0.08 | 0.00 | 0.08 | 0.03 | 0.03 | 0.14 | 1.00 |  |  |  |
| Religion | -0.06 | 0.02 | 0.00 | 0.01 | 0.01 | -0.04 | 0.00 | 0.00 | 0.06 | -0.04 | 0.00 | -0.02 | 1.00 |  |  |
| Caste | -0.02 | 0.12 | 0.00 | 0.03 | -0.02 | -0.09 | -0.11 | 0.23 | 0.15 | 0.01 | 0.17 | -0.02 | -0.10 | 1.00 |  |
| Living Arrangement | 0.03 | 0.05 | 0.20 | 0.19 | 0.65 | -0.05 | -0.07 | -0.12 | -0.08 | 0.07 | 0.04 | 0.00 | 0.01 | -0.01 | 1.00 |

Table S2. Variance Inflation Factor (VIF)

| Variable | VIF | 1/VIF |
| --- | --- | --- |
| Marital Status | 1.97 | 0.51 |
| Living Arrangement | 1.74 | 0.57 |
| Sex | 1.57 | 0.64 |
| Years of Schooling | 1.44 | 0.69 |
| Smoking | 1.31 | 0.76 |
| Alcohol | 1.29 | 0.78 |
| Age | 1.22 | 0.82 |
| Residence | 1.21 | 0.83 |
| No of chronic disease | 1.15 | 0.87 |
| Self-rated health | 1.12 | 0.89 |
| Caste | 1.19 | 0.84 |
| Wealth quintile | 1.1 | 0.91 |
| Region | 1.04 | 0.96 |
| Religion | 1.02 | 0.98 |
| Mean VIF | 1.31 |  |
